# Advanced Glycation End Products Induce Proliferation and Migration of Human Aortic Smooth Muscle Cells through PI3K/AKT Pathway

**DOI:** 10.1155/2020/8607418

**Published:** 2020-07-13

**Authors:** Gang Yuan, Guangyan Si, Qingchun Hou, Zhaonan Li, Kaiqiang Xu, Yuping Wang, Weiming Wang, Xiongfei Xu, Lei Zhang, Xiaolei Sun, Huqiang He, Hong Zeng, Yong Liu

**Affiliations:** ^1^Department of General Surgery (Vascular Surgery), The Affiliated Hospital of Southwest Medical University, Luzhou 646000, China; ^2^Department of Intervention, Traditional Chinese Medicine Hospital Affiliated to Southwest Medical University, Luzhou 646000, China; ^3^Department of Vascular Surgery, Zigong No.4 People's Hospital, Zigong 643000, China; ^4^Cardiovascular and Metabolic Diseases Key Laboratory of Luzhou, Luzhou 646000, China; ^5^Key Laboratory of Medical Electrophysiology, Ministry of Education & Medical Electrophysiological Key Laboratory of Sichuan Province (Collaborative Innovation Center for Prevention of Cardiovascular Diseases), Institute of Cardiovascular Research, Southwest Medical University, Luzhou 646000, China

## Abstract

Advanced glycation end products (AGEs) have been widely regarded as an important inducing factor in the pathogenesis of diabetic arteriosclerosis, and the proliferation and migration of vascular smooth muscle cells (VSMCs) are also involved in this process. However, it is not clear whether AGEs promote atherosclerosis by inducing the proliferation and migration of VSMCs. To figure out this question, this study investigated the effects of AGEs on the proliferation and migration of human aorta vascular smooth muscle cells (HASMCs) and the underlying mechanisms. This study evaluated the effects of different concentrations of AGEs on cell proliferation and migration. CCK8, transwell, and western blotting assays demonstrated that AGEs significantly increased cell proliferation and migration in a concentration-dependent manner and that the optimal proproliferative and promigratory concentrations of AGEs were 10 mg/L and 20 mg/L, respectively. AGE-induced cell proliferation, migration, and expression of filament actin (F-actin) were markedly attenuated by a PI3K inhibitor (LY2940002). Additionally, the phosphorylation of AKT was reduced when the receptor of advanced glycation end product (RAGE) gene was silenced by lentivirus transfection, which led to a concomitant reduction of the expression of proliferation and migration-related proteins. These data indicate that AGEs may activate the PI3K/AKT pathway through RAGE and thus facilitate the proliferation and migration of HASMCs.

## 1. Introduction

Diabetes mellitus (DM) is a major cardiovascular risk factor and is associated with increased cardiovascular events and mortality. Atherosclerosis (AS) is one of the most common vascular complications of diabetes mellitus and a leading cause of death and disabling cardiovascular disease [1, 2]. The underlying mechanisms of diabetic atherosclerosis can be attributed to a combination of factors, including oxidative stress, inflammation, increased expression of growth factors, and increased production of AGEs [3, 4]. AGEs are the products of nonenzymatic glycosylation and oxidation of a group of proteins and lipids after continuous contact with reducing sugars or short-chain aldehydes. They are common in the vasculature of diabetic patients, accelerating the development of AS. The interaction of AGEs with their receptors (RAGE) activates downstream molecular pathways leading to atherosclerosis [5]. A previous study reported that activated vascular smooth muscle cells (VSMCs) can effectively proliferate and migrate, promoting the repair of the vascular wall [6]. However, whether AGEs induce the proliferation and migration of VSMCs so as to promote AS is still unclear.

The migration and proliferation of VSMCs are essential in the process of vascular remodeling [7]. Under normal circumstances, VSMCs are mainly found in the middle membrane of the arteries and are major cellular components that maintain the morphology and function of blood vessels. Nevertheless, when intima is stimulated by inflammation, injury, stress, and other harmful stimuli, VSMCs proliferate rapidly and migrate to the injured site, participating in intimal hyperplasia [8]. Intimal hyperplasia is a pathogenic marker of restenosis after stent implantation, including abnormal proliferation and migration of VSMCs and accumulation of extracellular matrix, which eventually leads to lumen stenosis and even occlusion [9]. In addition, excess AGEs in diabetic patients accelerate the progression of arteriosclerosis by affecting arterial wall thickening. Interestingly, this effect is particularly marked by the proliferation and migration of VSMCs [10], which implied that the proliferation and migration of VSMCs may play an important role in the development of atherosclerosis.

Thus, this study is aimed at investigating the effects of AGEs on the proliferation and migration of HASMCs and revealing the molecular mechanisms underlying these effects.

## 2. Materials and Methods

### 2.1. Cell Culture and Identification

The primary human aorta vascular smooth muscle cells (HASMCs, ScienCell, California, USA) were cultured in Dulbecco's Modified Eagle Medium (DMEM, Hyclone Laboratories, Utah, USA) supplemented with 10% fetal bovine serum (FBS, ScienCell, California, USA), penicillin (100U/mL), and streptomycin (100 *μ*g/mL ) and then incubated at 37°C in a humidified 5% CO_2_ atmosphere. Smooth muscle actin (*α*-SMA) expression of the cells was identified by immunofluorescence assay. Cells that have been passaged for 3 to 5 generations were used.

### 2.2. Cell Proliferation

Cell proliferation was assessed using Cell Counting Kit-8 (CCK8, Dojindo, Shanghai, China). HASMCs were seeded into 96-well plates at 1000 cells/well in 200 *μ*L of medium for 24 h. The adherent cells were serum-starved for another 24 h and then treated with different concentrations of AGEs (5, 10, 20, and 40 mg/L; BioVision, Japan) or without any AGEs for 24 h in a humidified atmosphere with 5% CO_2_. Following the experimental treatment, supplemented with 10 *μ*L of CCK8 solution for each well, the cells were incubated at 37°C for 3 h. Finally, the absorbance value was measured at 450 nm using a microplate reader.

### 2.3. Cell Migration

Cell migration was assessed by transwell insert chambers (Corning, Maine, USA). HASMCs (5 × 10^3^ cells) were seeded in the serum-free medium into the upper chamber; the lower chamber was filled with DMEM with 10% FBS and supplemented with different interventions according to the experimental design. After 24 h, the cells from the upper surface of the filters were carefully wiped off with a cotton swab while those on the lower surface were washed with PBS, fixed for 30 min, air-dried at room temperature, and stained with 0.1% crystal violet (Beyotime Biotechnology, Shanghai, China) for 20 min. Cell migration was then visualized microscopically.

### 2.4. F-Actin Staining

HASMCs (5.0 × 10^3^ cells/mL) were inoculated uniformly on glass slides for 24 h. Synchronized cells were then subjected to one of the following treatments for 24 h: AGEs (0 mg/L) which represent the control group, AGEs (20 mg/L), or AGEs (20 mg/L) + LY294002 (a commonly used PI3K inhibitor, Selleck, USA). Cell slides were washed with PBS, fixed for 30 min, washed again, permeabilized in 0.2% Triton X-100 at room temperature for 5 min, treated with 1% bovine serum albumin for 30 min, stained with 10 *μ*g/mL FITC-rhodamine phalloidin (AmyJet, Wuhan, China) at room temperature for 30 min, washed again, treated with 50 *μ*g/mL RNase at 37°C for 30 min, stained with 10 *μ*g/mL propidium iodide (PI, BD, USA) for 30 min, washed again, and finally sealed with 50% buffer glycerin. F-Actin staining was visualized and analyzed by confocal microscopy.

### 2.5. RAGE Knockdown by RAGE-RNAi Lentivirus

RAGE-RNAi lentivirus vector construction and packaging was entrusted to Shanghai Genechem Co. Ltd. The siRNA target sequence was 5′-AGTCCGTGTCTACCAGATT-3′, and the contrast insertion sequence was 5′-TTCTCCGAACGTGTCACGT3′. The MOI of HASMCs was found to be 100 through a preliminary experiment following the manufacturer's instructions. Cells were seeded on 24-well plates (3 × 10^4^ cells/well) until they reached 70% confluence, at which time they were cultured with prediluted RAGE-RNAi lentivirus and 8 *μ*g/mL polybrene at 37°C under 5% CO_2_ for 8 h. Cell growth was observed, after which cells were maintained in fresh medium for 96 h at which time virus infection was visualized using inverted fluorescence microscopy. When the GFP expression exceeded 90%, the expression of RAGE protein was detected by western blotting.

### 2.6. Western Blotting

Briefly, whole cell lysates were extracted from cultured HASMCs with Radioimmunoprecipitation Assay (RIPA) buffer (Beyotime Biotechnology, Shanghai, China) and the protein concentration determined using the BCA method as recommended by the manufacturer (Beyotime Biotechnology, Shanghai, China). The collected proteins were boiled for 5 min and separated using 10% SDS-polyacrylamide gels (SDS-PAGE; Sigma, USA), then transferred to PVDF membranes (Millipore, Bedford, MA, USA) using 10% SDS-PAGE gel electrophoresis. Next, samples were sealed with 5% nonfat milk powder and TBST at room temperature for 1 h, after which membranes were incubated with the following primary antibodies: rabbit anti-human PCNA (1 : 1000), Cyclin-D1 (1 : 1000), MMP-9 (1 : 800), MMP-2 (1 : 500), RAGE (1 : 800), AKT (1 : 1000), phospho- (p-) AKT (1 : 2000), and antibody GAPDH (1 : 5000) (CST, Santa Cruz, Biotechnology, CA, USA) at 4°C overnight. Samples were then washed 3 times with 10 mL of TBST (15 min), after which the PVDF membranes were incubated with HRP-labeled goat anti-rabbit IgG (1 : 5000) (Abgent, Suzhou, China) at 37°C for 1 h. Finally, the specific protein bands were visualized with an ECL Plus kit (Beyotime Biotechnology, Shanghai, China) and quantified with the Quantity One software (Bio-Rad, USA).

### 2.7. Statistical Analysis

All data were presented as means ± standard deviation (SD) and analyzed using statistical software SPSS19.0. Comparisons between the groups were performed by one-way analysis of variance (ANOVA). Student's *t*-tests were conducted to determine the differences between two groups. *P* < 0.05 was considered as significant difference.

## 3. Results

### 3.1. HASMC Identification

**Figure 1:**
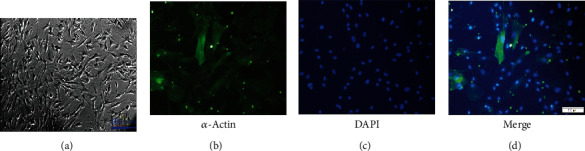
Identification of HASMCs. (a) Cell morphological images taken with an inverted phase contrast microscopy under 100-fold objective. (b) Image of immunofluorescence-stained cells taken with a fluorescence microscope under 200-fold objective. (c) Image of cells with nuclear staining. (d) Merged image of (b) and (c).

The immunofluorescence imaging (Figure 1) revealed that the HASMCs expressed *α*-SMA, as the positive green fluorescence was emitted from the cytoplasm of the cells.

### 3.2. Effects of AGEs on Cell Proliferation and Migration

**Figure 2:**
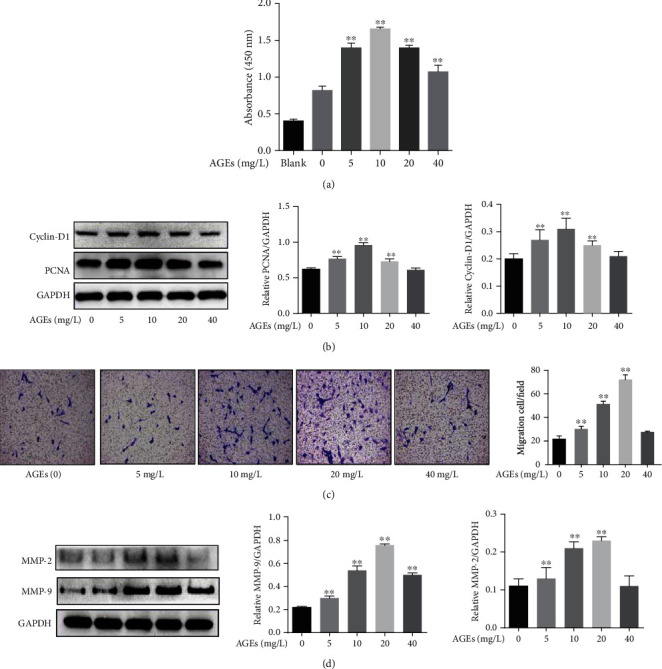
Effects of AGEs on HASMC proliferation and migration. (a) Optical density at 450 nm, measured in the CCK8 assay, following treatment with AGEs (5,10, 20, and 40 mg/L) for 24 h (*N* = 6 in each group). (b) Effect of AGEs on the expression of PCNA and Cyclin-D1 (PCNA and Cyclin-D1 are associated with cell proliferation, GAPDH was used as a loading control, *N* = 3 in each group). (c) Migration of the HASMCs treated with AGEs (5,10, 20, and 40 mg/L) for 24 h (*N* = 5 in each group). (d) Effect of AGEs on the expression of MMP-9 and MMP-2 (MMP-9 and MMP-2 are associated with cell migration, GAPDH was used as a loading control, *N* = 3 in each group). ^∗∗^*P* < 0.01 was defined as significant difference.

CCK8 assay showed that, after HASMCs were treated with AGEs, the proliferation ability of cells was significantly enhanced, compared with control cells, in a concentration-dependent manner, and cell proliferation ability was the strongest when AGE concentration was 10 mg/L (Figure 2(a)). AGE-induced HASMC migration was detected by the transwell assay; the results demonstrated that, in a certain range, AGEs promoted the migration of cells in a concentration-dependent manner, and the promigratory ability of AGEs increased as the AGE concentration increased, peaked at 20 mg/L, and then decreased gradually (Figure 2(c)), compared with those of the control cells. In addition, as shown in Figures 2(b) and 2(d), the expression of proteins related to proliferation and migration showed concentration-dependent changes to AGEs.

### 3.3. AGEs Activated PI3K/AKT Signaling

**Figure 3:**
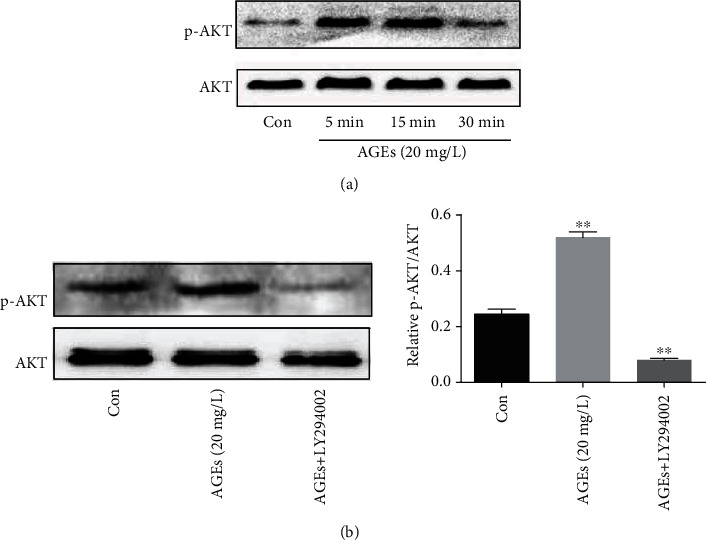
Phosphorylation of AKT induced by AGEs. (a) AGE (20 mg/L) treatment of cells significantly increased phosphorylation of AKT levels at 5 min to 15 min compared to control cells (*N* = 3 in each group). (b) p-AKT induced by AGEs was inhibited by LY294002 (PI3K inhibitor) (*N* = 3 in each group). ^∗∗^*P* < 0.01 was defined as significant difference.

**Figure 4:**
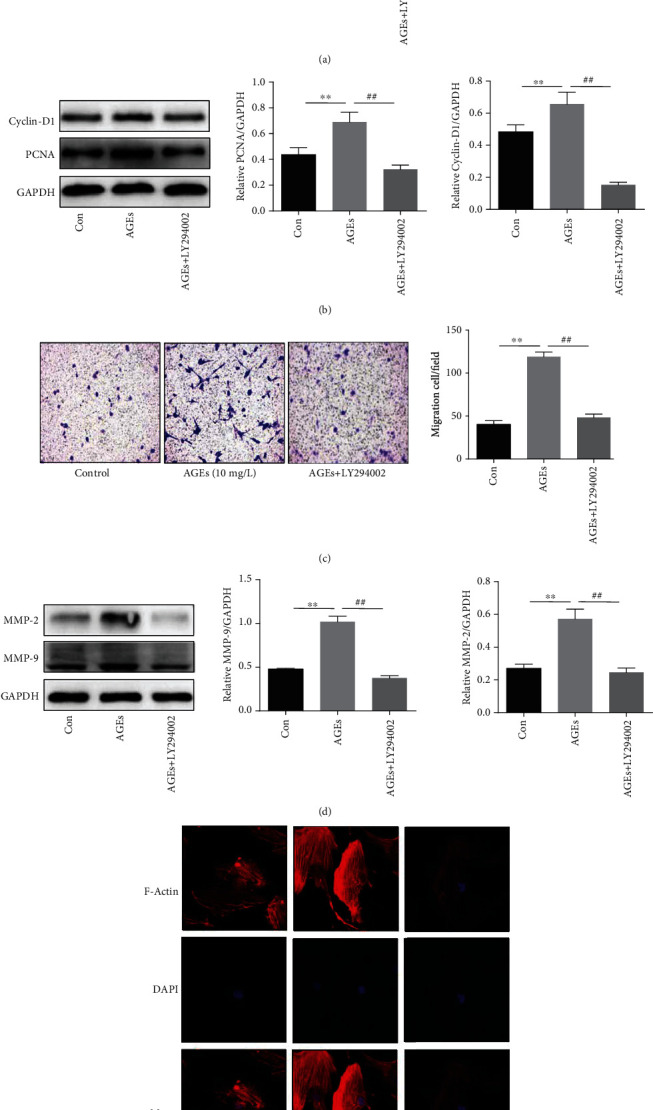
Effects of AGEs on the proliferation and migration of HASMCs through the PI3K/AKT pathway. (a) Optical density at 450 nm, measured by CCK8 assay; HASMCs were pretreated with and without LY294002 (20 *μ*M) and then treated with AGEs (10 mg/L) for 24 h (*N* = 6 in each group). (b) The expression of PCNA and Cyclin-D1 (PCNA and Cyclin-D1 are associated with cell proliferation, GAPDH was used as a loading control, *N* = 3 in each group). (c) Migration of the HASMCs pretreated with and without LY294002 (20 *μ*M) and then treated with AGEs (20 mg/L) for 24 h (*N* = 5 in each group). (d) The expression of MMP-9 and MMP-2 (MMP-9 and MMP-2 are associated with cell migration, GAPDH was used as a loading control, *N* = 3 in each group). (e) F-Actin expression was measured through cytoskeleton staining (FITC-rhodamine phalloidin) and observed using a laser confocal scanning microscope. ^∗∗^*P* < 0.01 was defined as significant difference. ^##^*P* < 0.01 was defined as significant difference compared with the AGE group.

In order to verify whether the PI3K/AKT signal pathway is involved in the proliferation and migration of HASMCs induced by AGEs, we detected the expression of AKT and its phosphorylated proteins by western blotting. We found that p-AKT levels were notably increased, compared to the control group, after cells were treated with AGEs (20 mg/L) for 5-15 min (Figure 3(a)). Phosphorylation of AKT induced by AGEs was significantly inhibited by LY294002 which is a potent protein kinase inhibitor of phosphotidylinsitol-3-kinase (PI3K); however, the total AKT levels were not obviously decreased (Figure 3(b)). To further investigate this effect, HASMCs were pretreated with and without LY294002 and then treated with AGEs; the results showed that the proliferation and migration of HASMCs induced by AGEs were dramatically inhibited by LY294002 (Figures 4(a)–4(d)). In addition, as shown in Figure 4(e), LY294002 also markedly reduced the expression of filament actin (F-actin) in HASMCs compared to the control cells.

### 3.4. RAGE-Mediated Activation of PI3K/AKT Signaling

**Figure 5:**
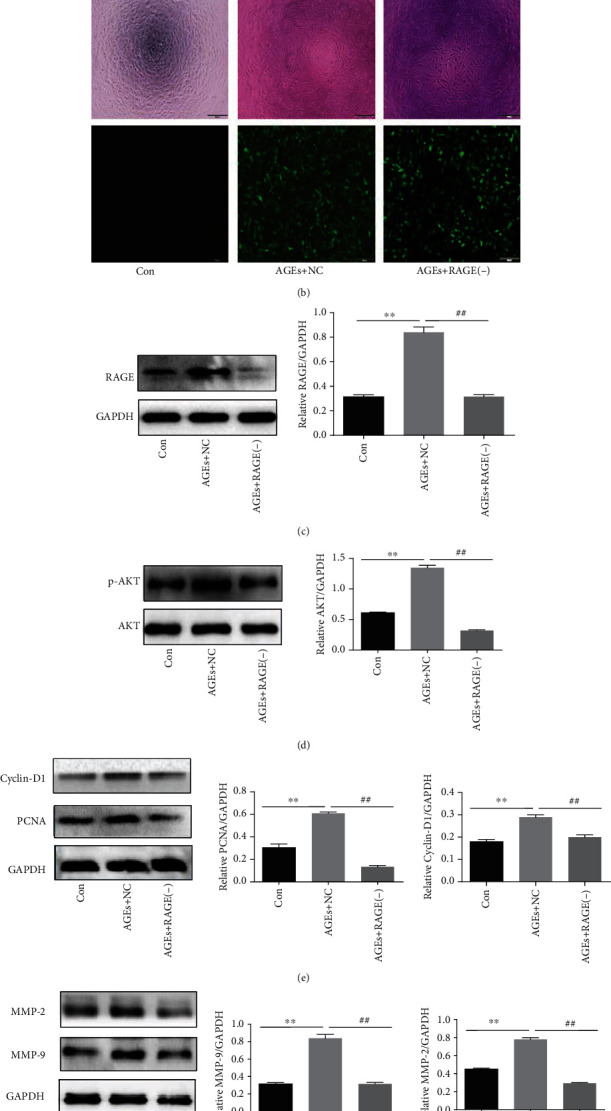
RAGE mediated activation of PI3K/AKT signaling pathway. (a) Effect of AGEs on the expression of RAGE (*N* = 3 in each group). (b) Images showing HASMCs infected with empty vector lentivirus (AGEs+NC) and lentivirus-RAGE-RNAi (AGEs+RAGE(-), NC means negative control); upper: brightfield, lower: fluorescence imaging of GFP expression. Magnification: 20-fold. (c) RAGE expression was detected by western blotting after RAGE-RNAi lentivirus transfection (*N* = 3 in each group). (d) The expression of phosphorylate AKT and total AKT levels in HASMCs after RAGE-RNAi lentivirus transfection (*N* = 3 in each group). (e, f) The expression of PCNA, Cyclin-D1, MMP-9, and MMP-2 in HASMCs after RAGE-RNAi lentivirus transfection (*N* = 3 in each group). GAPDH was used as a loading control, ^∗∗^*P* < 0.01 was defined as significant difference compared with the control, and ^##^*P* < 0.01 was defined as significant difference compared with the AGEs+negative control.

RAGE is a transmembrane protein, which mainly mediates the transmission of the intracellular signal.  Figure 5(a) shows that the expression of the RAGE protein in HASMCs is incrementally dependent on AGE concentration. To investigate whether RAGE mediates PI3K/AKT signaling, the RAGE gene in HASMCs was silenced through RAGE-RNAi lentivirus vector transfection. Compared with the control group, a large amount of green fluorescence was stably detected in both the empty vector lentivirus transfection group and the RAGE-RNAi lentivirus transfection group (Figure 5(b)), indicating successful transfection. Further, we found that the expression of RAGE was dramatically downregulated after the RAGE gene was knocked down (Figure 5(c)). Interestingly, the expression of phosphorylated AKT was also inhibited significantly, while the expression of total AKT was almost unchanged (Figure 5(d)). As shown in Figures 5(e) and 5(f), compared with cells in the control group, the expression of proteins related to proliferation and migration of HASMCs was notably reduced after RAGE was silenced, revealing that the proliferation and migration of HASMCs was also significantly inhibited after RAGE was blocked.

## 4. Discussion

AS is a chronic and complicated process involving numerous types of cells and cell-to-cell interactions that ultimately lead to progression from the “fatty streak” to formation of more complex atherosclerotic plaques [11]. In the early stage, damage to the vessel intima can initiate a series of self-repair, but excessive proliferation and migration of VSMCs accelerate progression of AS [12]. Studies found that AGEs largely exist in the arterial lipid stripes, atherosclerotic plaques, and macrophages of DM patients, the quantity of which is positively related to the severity of atherosclerotic plaques [13–16], which means AGEs can accelerate the development of AS in diabetics. Therefore, the study of the effect of AGEs on the proliferation and migration of VSMCs and its related mechanism is significant for the prevention and treatment of diabetic arteriosclerosis.

Although some studies suggested that AGEs can induce the proliferation of VSMCs and play an important role in the pathogenesis of AS in DM patients [17–20], the effect of different AGE concentrations on cell proliferation and migration remains unclear. When studying the effect of AGEs-BSA on the proliferation of rabbit VSMCs in vitro, Satoh et al. found that AGEs-BSA could significantly promote the proliferation of rabbit VSMCs in the range of 1-10 mg/L, and the proliferation was notably inhibited when the concentration exceeded 20 mg/L [21]. This study further confirmed the relationship between the proliferation and migration of HASMCs and the concentration of AGEs. It was found that cell proliferation and migration predominated when the AGE range was low (5-20 mg/L), but decreased significantly when the concentration increased to 40 mg/L (Figures 2(a) and 2(c)). In addition, our previous study revealed that AGEs could promote apoptosis in HASMCs within the range of 50~200 mg/L and induce calcification in a concentration-dependent manner [22]. The mechanism of different cellular effects of different AGE concentrations is not clear at present, but we speculate that this may be partly due to the fact that high concentrations of AGEs induce apoptosis and inhibit the activity of cell proliferation and migration.

A study showed that the expression of PCNA, Cyclin-D1, MMP-2, and MMP-9 genes can induce the formation of colorectal cancer and promote cancer cell proliferation, migration, and invasion [23], suggesting that these genes may be closely related to cell proliferation and migration. Some other studies reported that PCNA and Cyclin-D1 mainly regulate cell proliferation and that MMP-2 and MMP-9 play important roles in cell migration and invasion [24, 25]. In this study, we observed that AGEs can upregulate the expression of PCNA, Cyclin-D1, MMP-2, and MMP-9 proteins (Figures 2(b) and 2(d)), which further proved that AGEs can activate the expression of these genes and induce HASMC proliferation and migration.

Now that it has been firmly confirmed that AGEs exert proproliferative and promigratory functions on VSMCs, it is necessary to identify the specific mechanism governing the process. Yoon et al. [26] confirmed that AGEs could initiate their proproliferative function on VSMCs by activating the pathways on which P38 and ERK are dependent. The PI3K/AKT pathway has been found having the function of regulating multiple target proteins to participate in cell proliferation and migration [9, 12, 27]. Shi et al. [28] reported that Irisin stimulates cell proliferation and invasion by targeting the PI3K/AKT pathway in human hepatocellular carcinoma. However, whether the PI3K/AKT pathway is involved in the proliferation and migration of VSMCs in patients with diabetes needs further verification. In this study, AGE-induced proliferation and migration of HASMCs were detected after the PI3K/AKT pathway was inhibited by LY294002. The results revealed that inhibition of the PI3K/AKT pathway not only reduced cell proliferation and migration activity (Figures 4(a) and 4(c)) but also downregulated the protein expression related to cell proliferation and migration (Figures 4(b) and 4(d)). In addition, this study also found that the expression of F-actin, the main cytoskeleton system involved in cell contraction, adhesion, and migration [29], decreased after inhibition of PI3K/AKT (Figure 4(e)). These results confirmed that the PI3K/AKT pathway plays an important role in the proliferation and migration of HASMCs induced by AGEs.

RAGE is a multiligand receptor member of immunoglobulin superfamily cell surface molecules. As transmembrane proteins on the cell membrane, RAGE and AGEs can initiate intracellular signal transduction through receptor-ligand binding [30]. In this study, the expression of the RAGE protein was confirmed to be positively dependent on AGE concentrations (Figure 5(a)), suggesting that AGEs may play biological roles through specific binding to RAGE. A previous study reported that lysophosphatidic acid is involved in tumor and angiogenesis through the RAGE and the AKT signal [31]. However, it is unclear whether AGE/RAGE, as a classical ligand-receptor binding axis, promotes the proliferation and migration of HASMCs by activating the PI3K/AKT pathway. In our experiments, the RAGE-RNAi lentivirus vector was transfected into HASMCs to silence the RAGE gene (Figures 5(b) and 5(c)). Subsequently, western blotting assay detection showed that AGEs induced AKT phosphorylation by RAGE (Figure 5(d)), which accelerated cell proliferation and migration (Figures 5(e) and 5(f)).

In conclusion, this study reveals that AGEs induce the proliferation and migration of HASMCs *in vitro*. It also confirms, at least in part, that the AGE/RAGE axis can activate the PI3K/AKT pathway and thus facilitates HASMC proliferation and migration. This implies that the PI3K/AKT signaling pathway plays a pivotal role in AGE-induced proliferation and migration of HASMCs. Therefore, reducing AGEs and the expression of RAGE or developing more effective drugs to inhibit the PI3K/AKT pathway may become a new strategy for the treatment of diabetic atherosclerosis. However, it has to be said that since this experiment only studied HASMCs cultured *in vitro*, it is necessary to conduct additional experiments *in vivo*.

## Data Availability

The data used to support the findings of the study are available from the corresponding author upon request.
